# Amino-Li-Resin—A Fiber Polyacrylamide Resin for Solid-Phase Peptide Synthesis

**DOI:** 10.3390/polym14050928

**Published:** 2022-02-25

**Authors:** Damilola C. Akintayo, Beatriz G. de la Torre, Yongfu Li, Fernando Albericio

**Affiliations:** 1 Peptide Science Laboratory, School of Chemistry and Physics, University of KwaZulu-Natal, Westville, Durban 4000, South Africa; damilolaakintayo141@gmail.com; 2 KwaZulu-Natal Research Innovation and Sequencing Platform (KRISP), School of Laboratory Medicine and Medical Sciences, College of Health Sciences, University of KwaZulu-Natal, Durban 4041, South Africa; 3 Biotide Core, LLC, 33815 SE Eastgate Circle, Corvallis, OR 97333, USA; mail@biotide-core.com; 4 Institute for Advanced Chemistry of Catalonia (IQAC-CSIC), Jordi Girona 18-26, 08034 Barcelona, Spain; 5 CIBER-BBN, Networking Centre on Bioengineering, Biomaterials and Nanomedicine, Department of Organic Chemistry, University of Barcelona, Martí i Franqués 1-11, 08028 Barcelona, Spain

**Keywords:** amino-Li-resin, solid-phase peptide synthesis, polyacrylamide, fibers

## Abstract

Amino-Li-resin is a new and unique polyacrylamide resin presented in the form of fibers and is found to be well suited for solid-phase peptide chemistry. Although amino-Li-resin swells much better in polar solvents, it is also compatible with some non-polar solvents. It comes with a high loading of functional amino groups, thus maximizing its productivity in terms of the amount of peptide per gram of resin. In addition to its mechanical stability, this resin shows excellent stability in basic and acidic reagents; thus, allowing its broad applicability for the synthesis of a wide range of biomolecules. Finally, the appropriateness of amino-Li-resin for solid-phase peptide synthesis (SPPS) has been demonstrated for the synthesis of several model peptides, including difficult sequences and those containing hindered amino acids, all of which afforded excellent crude purity, as shown by high-performance liquid chromatography (HPLC) analysis.

## 1. Introduction

The pioneering work of Bruce Merrifield [1], a Nobel Laureate, in developing a brilliant methodology for the synthesis of small to medium/large peptides on a laboratory and industrial scale [2,3] has brought about untold possibilities for the application of peptides in science. This approach, known as solid-phase peptide synthesis (SPPS), effectively allows the preparation of peptides by anchoring the carboxylic end of an N-protected amino acid to a resin/solid support using excess reagents. The method involves a repetitive cycle of deprotection, coupling of successive amino acids in the C-N direction, and washing of unused reagents and by-products by filtration [4]. The combined use of excess reagents and the straightforward workup are key for the superior effectiveness of the strategy, as reflected by the excellent purity (≥95%) of the crude peptides in a very short period.

The SPPS process is holistic and, therefore, its success depends on several factors, such as the resin [5,6,7], solvent (taking into consideration its polarity and viscosity) [8,9], coupling reagents [10,11,12], and protection scheme [13,14,15,16,17]. Despite the immense amount of work done over the years to improve these factors and thus facilitate the development of any kind of sequence, some “difficult peptide sequences” remain challenging [18,19]. In this regard, research efforts are ongoing to circumvent the many concerns that have arisen. The field has witnessed a considerable number of improvements, with the resin makeup being a major driving force. The resin, which contains a linker grafted onto an insoluble polymeric solid support, is critical for the effectiveness of any given synthetic procedure as it is central to the performance of the other factors.

Industrially, polystyrene (PS)-based resins cross-linked with 1% of divinylbenzene is the solid support most widely used for SPPS. Polystyrene swells well in dichloromethane (DCM), the first solvent used in the seminal work of Merrifield. However, this solid support shows limited swelling capacity in polar solvents, which usually perform better for the synthesis of large peptides and there is a need to replace the hazardous DCM. More so, the high hydrophobicity of PS resulting from the high number of aromatic rings can cause π–π interactions with other aromatic rings of the same resin or with those of the trityl protecting group, leading to extra cross-linking, thereby reducing the accessibility of reactive sites.

To overcome this drawback, PS resins with different cross-linking and, more importantly, different solid supports based on polyamides, polyacrylates, polyethylene glycol (PEG), and those obtained by grafting two of these materials were proposed to improve their applicability for a wide range of peptide targets and allow the use of other solvents, mostly green ones.

In 1973, Sheppard was the first to propose the massive use of dimethylformamide (DMF) as the solvent of choice for SPPS since this reagent resembles the growing peptide bond. In an attempt to change the paradigm (PS and DCM), he proposed that the joint use of DMF and a polar resin would most likely bring about a greater improvement in SPPS [20]. In this regard, his group embarked on the preparation of the first polydimethylacrylamide support by reacting dimethylacrylamide, 1,2-ethanediacrylamide, and a functionalized acrylamide-based monomer [21,22,23]. Fully beaded resins were obtained with a more hydrophilic monomer through a demarcated suspension polymerization methodology [22,24]. Several other acrylamide-based supports have since appeared in the literature for SPPS [25,26]. Worthy of note is the polyacrylamide resin (SPAR-50) developed by Sparrow [27,28]. SPAR-50 swells well in polar protic solvents and has been reported to produce peptides with high purity and better yields. However, its commercialization was later discontinued, possibly due to safety concerns as the synthetic process requires the use of CCl_4_ or DCM and other hazardous chemicals. Hence the need exists to revive the use of polyacrylamide-based resins using a greener approach (vide infra p. 4).

In the mid-1980s, two independent groups proposed poly(ethylene glycol)-PS-based resins designated PEG-PS by Barany, Albericio, and Zalipsky [7,29] and TentaGel by Bayer and Rapp [30,31]. PEG-PS was obtained by amide formation between carboxylic acid-containing PEGs and amino-PS resins, whereas TentaGel was made by grafting PEG on PS beads. The superior performance of these two resins conferred by the addition of a hydrophilic polyether like PEG to the hydrophobic core of PS makes them compatible with polar and non-polar solvents. This feature has fueled the development of other PEG-PS resins, which, in most cases, have also been commercialized. Thus, Champion I and II (designated as Novagel) were prepared by the incorporation of linear blocks and branched-chain PEGs, respectively [32]. ArgoGel was obtained by attaching PEG to branched diol supports [33], OctaGel has its sites capped with PEG and the surface solely containing the reactive site, thus giving it a high degree of uniformity [5].

The modification of PEG on the PS core in all these resins tends to confer relatively uniform swelling in a variety of solvents, ranging from toluene to H_2_O, and good performance for the synthesis of a broad number of peptides. However, these modified resins have the following disadvantages: (i) low loading (0.2–0.4 mmol/g); (ii) leaching: and (iii) high cost. PEG is usually linked to the PS by an acid-labile bond, which can be released during the final acidic cleavage step. This release will compromise the purity or content of the final peptide because PEG will also precipitate during the work-up with ether. The modification process adds to the manufacturing complexity and cost due to its small production scale. Moreover, industrial applications require the use of large batches (multi-Kgs) of resins, which are often extremely costly.

PEG-based resins containing minute quantities of functionalities, such as polyamide, PS [34,35,36], and acrylate with polymerizable vinyl moiety [37,38,39], have been reported. PEGA is a flow-stable and highly branched PEG-based resin prepared by the copolymerization of bisacrylamidoprop-1-yl-PEG_1900_, 2-acrylamidoprop-1-yl[2-aminoprop-1-yl]-PEG_300_, and *N*,*N*-dimethyl acrylamide. This highly polar support prepared by a simple synthetic procedure using inexpensive starting material was designed to facilitate peptide solvation for the synthesis of long peptides. Cross-linked-ethoxylate-acrylate-resin (CLEAR) is another highly cross-linked PEG-based resin prepared either by bulk or suspension processes and involving the copolymerization of trimethylolpropane ethoxylate cross-linkers with readily available monomers in a single step. This class of solid support demonstrated good swelling properties in common solvents and has been reported to be highly stable for SPPS protocols. However, it may be unstable in specific strong bases.

Eventually, Meldal and his group [40,41] and Cote [6,42] independently reported a completely PEG-based resin, namely ChemMatrix (CM). CM has been widely used in SPPS, especially for long and difficult peptides [43,44,45]. The vicinal arrangement of carbon-oxygen bonds throughout the chain makes the resin amphiphilic and, as such, it is well-solvated in a wide range of solvents. CM is superior to other reported resins in terms of the purity of the final peptide, mechanical strength, and chemical stability in diverse reagents. However, it is expensive and difficult to produce on a large scale. Furthermore, it has got a poor recovery, and it requires extra solvents to drive the reaction to completion.

To facilitate the solid-phase synthesis of a large range of peptides and at the same time overcome the issues associated with the resins already on the market, we have continuously pursued new polar resins with good swelling capacity (≥4 mL/g) in a broad number of solvents [8,46]. In our endeavors, we studied the second generation of amino-polyacrylamide resin (amino-Li-resin) designed and developed by Yongfu Li.

In this regard, we examined the swelling capacity, morphology, chemical stability, and synthetic performance of this resin for the synthesis of several model peptides. To this end, Fmoc-Rink-Amide-Li-resin was used to synthesize the following sequences, which were encountered as difficult sequences or sequences containing hindered amino acids: leu-enkephalin (H-YGGFL-NH_2_); Ile^2^_,_Ile^3^-leu-enkephalin (H-YIIFL-NH_2_); RGD peptide (H-RGDfK-NH_2_); ^66^Gly-^65−74^ACP (H-VGAAIDYING-NH_2_), and a modified decaalanine (H-AAAAAAAAAAKKK-NH_2_) to test its utility.

## 2. Experimental Section

### 2.1. Materials and Methods

Amino-Li-resin was provided by Biotide Core, LLC, Corvallis, OR, USA. All reagents and solvents for this study were obtained commercially and used without further purification. Piperidine, acetonitrile (HPLC grade), dimethyl formamide and other organic solvents used were purchased from Merck Life Science (Pty) Ltd., Modderfontein, South Africa; and the Fmoc amino acids were obtained from Iris Biotech, Marktredwitz, Germany. Infrared (IR) spectra were obtained on a PerkinElmer Universal ATR spectrum 100 Fourier transform infrared (FT-IR) spectrometer. The high-performance liquid chromatography (HPLC) analysis was conducted on a reversed-phase C_18_ column (4.6 mm × 150 mm, 3.6 µm) at a flow rate of 1.0 mL/min, using linear gradients of 4.5 × 10^−2^% Trifluoroacetic acid (TFA) and 3.6 × 10^−2^% acetonitrile (ACN). High-performance liquid chromatography/mass spectrometry (HPLC/MS) was also obtained on a reversed-phase C_18_ column (4.6 mm × 150 mm, 3.6 µm) by means of aqueous (0.1%) formic acid and formic acid (0.07%) in can, as eluents.

### 2.2. Swelling Capacity

0.2 g of resin was added to a 5 mL syringe fitted with a 0.45 µm filter and furnished with sufficient solvent to give a final volume of 5 mL for 5–10 min to allow the resin to swell to maximum capacity. The piston of the syringe was used to compress the swollen resin to completely drain off the solvent, after which it was carefully drawn until the resin recovered its highest volume, which was then measured. By averaging the void volume of the tip and the syringe to be 0.15 mL, the swelling was determined using Equation (1):

(1) $$\mathrm{Swelling}\;\left(\mathrm{mL}/g\right)=\frac{volume\;of\;the\;swelled\;resin-0.15\;\mathrm{mL}}{0.2\;g}$$

### 2.3. Preliminary Washing

Amino-Li-resin was subjected to preliminary treatment by washing three times using 5% *N*,*N*-diisopropylethylamine (DIEA)/DCM (1 min) to neutralize any acid present. Afterwards, it was washed twice with DCM (1 min) and finally washed thrice with DMF (1 min) before it was used for the synthesis of the model peptides.

### 2.4. Peptide Synthesis

All operations involving the manual synthesis and peptide elongation were done in polypropylene syringes equipped with a permeable polyethylene disk. The synthetic protocol adopted involves the removal of soluble chemical reagents and all solvents through suction. The manual synthesis of the chosen model peptides was done using Fmoc-Rink-amide Li-resin (0.1 mmol scale, loading 0.8 mmol/g) at ambient temperature. The deprotection step (Fmoc removal) was carried out using 20% piperidine in dimethylformamide (1 × 1 min, 2 × 10 min), while other intermediate washing steps between each amino acid coupling and subsequent Fmoc removal was accomplished with dimethylformamide (5 × 1 min) and dichloromethane (5 × 1 min) using 10 mL of solvent per gram of resin. At room temperature, the peptidyl resin was dried under vacuum and subjected to a global deprotection and cleavage for 60 min using a freshly prepared cocktail (95% trifluoroacetic acid (TFA): 2.5% tri-isopropylsilane (TIS): 2.5% H_2_O). The crude peptide was crashed out using cold diethyl ether. The solution was then centrifuged, and the solvent was carefully decanted. The precipitation and cleaning processes were repeated three times, after which, the crude peptides were analyzed by HPLC and HPLC/MS using the following HPLC gradients: 5–60% B into A in 15 min for H-Tyr-Gly–Gly-Phe-Leu-NH_2_ and H-Tyr-Ile–Ile-Phe-Leu-NH_2_, 5–95% B into A in 15 min for H-Arg-Gly-Asp-DPhe-Lys-NH_2_, H-Val-Gly-Ala-Ala-Ile-Asp-Tyr-Ile-Asn-Gly-NH_2_, and H-Ala-Ala-Ala-Ala-Ala-Ala-Ala-Ala-Ala-Ala-Lys-Lys-Lys-NH_2_.

## 3. Results and Discussion

### 3.1. Preparation

**Figure 1:**
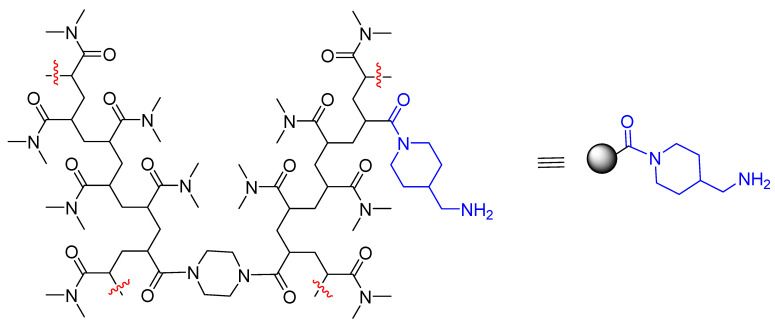
Chemical structure of amino-Li-resin prepared from *N*,*N*-dimethylacrylamide as a monomer, *N*,*N*’-bis(acryloyl)piperazine as cross-linker, 1-[1-(*N*-acrylyl)piperidin-4-yl]methanamine as functionalizing moiety, and *N*,*N*,*N*’,*N*’-tetramethylethylenediamine as initiator.

Amino-Li-resin was prepared by polymerization using *N*,*N*-dimethylacrylamide as a monomer, *N*,*N*’-bis(acryloyl)piperazine as cross-linker, 1-[1-(*N*-acrylyl)piperidin-4-yl]methanamine as functionalizing moiety, and *N*,*N*,*N*’,*N*’-tetramethylethylenediamine (TEMED) as initiator [47], following an *in house* proprietary fiberization technology (Figure 1). The use of a rather high proportion of *N*,*N*’-bis(acryloyl)piperazine makes the final resin highly cross-linked, thus conferring high mechanical stability. The proportions of the different reagents were calculated to afford a loading of 0.8 mmol of amino per gram of resin.

An advantage of the amino-Li-resin in comparison with amino-PS resins is that the 4-(aminomethyl)piperidin-1-yl functionalization, an aliphatic amine, is more reactive than the benzylamine or benzhydrylamine in the case of PS resins. This is important because the first reaction involves the incorporation of a linker, which is often an expensive reagent, through an amide bond. Furthermore, this amide bond will be more acid-stable than the amide bond formed by the same linker and amino-PS resins, thus preventing the formation of side products during the final global deprotection and cleavage step [48].

### 3.2. Physical Properties

**Figure 2:**
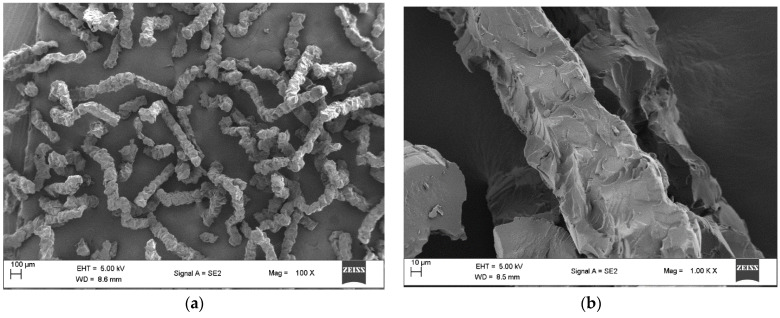

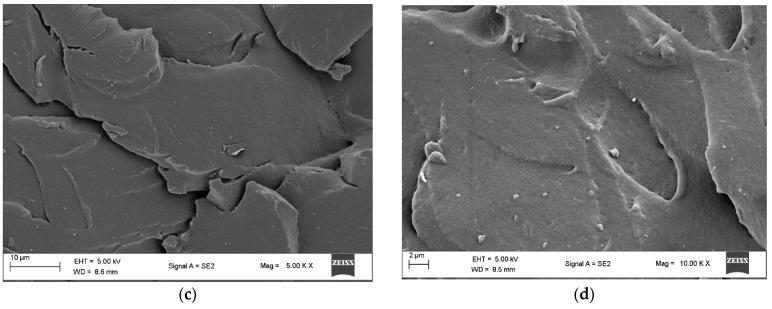
SEM images of amino-Li-resin: (**a**) 100 µm (100 X), (**b**) 10 µm (1.00 K X), (**c**) 10 µm (5.00 K X), and (**d**) 2 µm (Mag = 10.00 K X).

**Figure 3:**
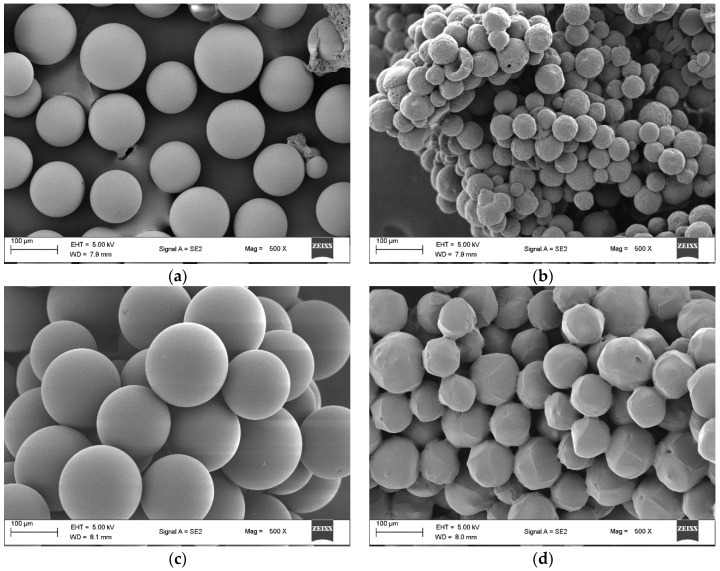
SEM images of (**a**) PS, (**b**) PEG-PS, (**c**) CLEAR, and (**d**) CM taken at 100 µm and magnification of 500.

Scanning electron microscope (SEM) images of amino-Li-resin (Figure 2a–d) confirmed the formation of fibers as different from the regular spherical beads observed with prominent resins like PS, PEG-PS, CLEAR, and CM (Figure 3a–d). Spherical beads are normally between 150 to 300 microns in diameter, whereas the fiber of amino-Li-resin is 150 microns in diameter and about 400 microns in length. The SEM images of amino-Li-resin fiber revealed a porous microstructure with a larger surface area, allowing for superior penetration of reagents and solvents over the beaded resins.

**Figure 4:**
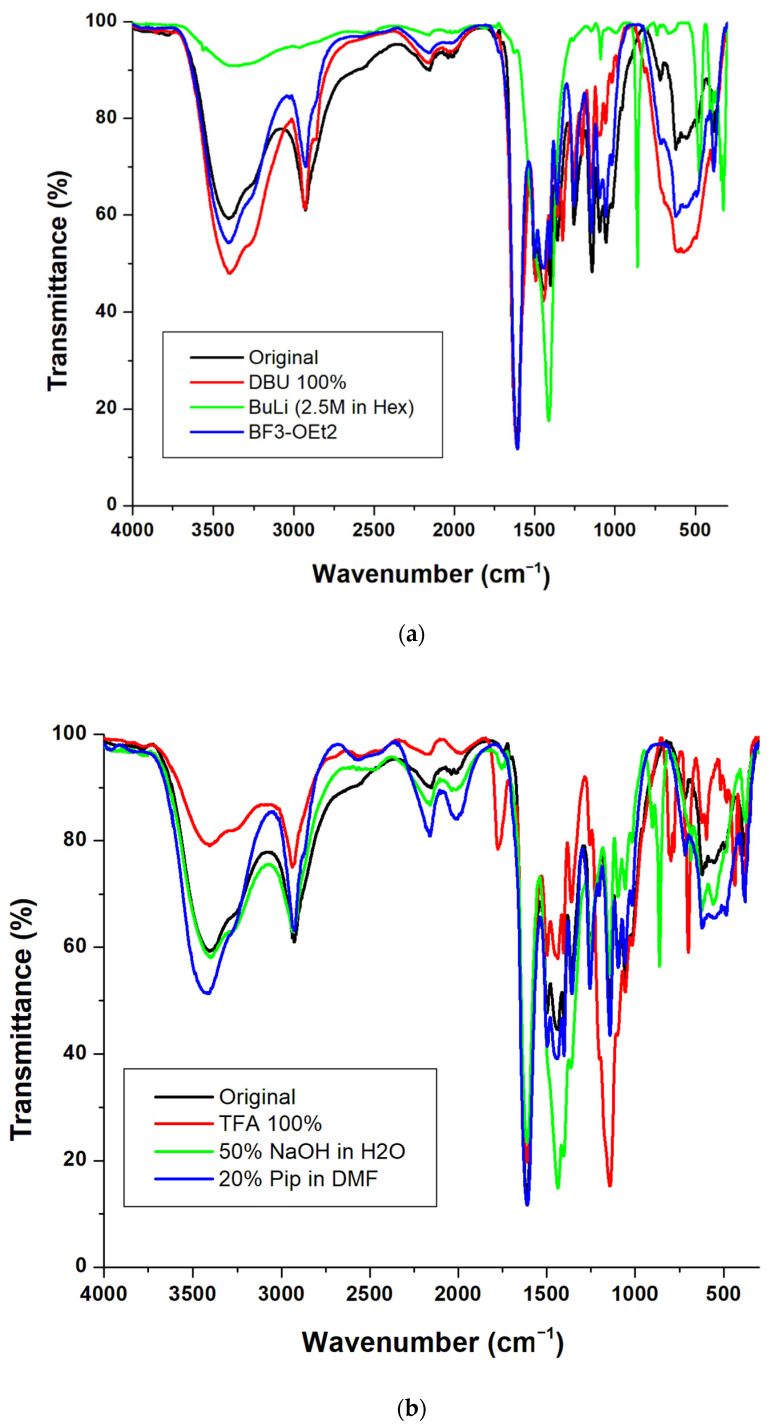

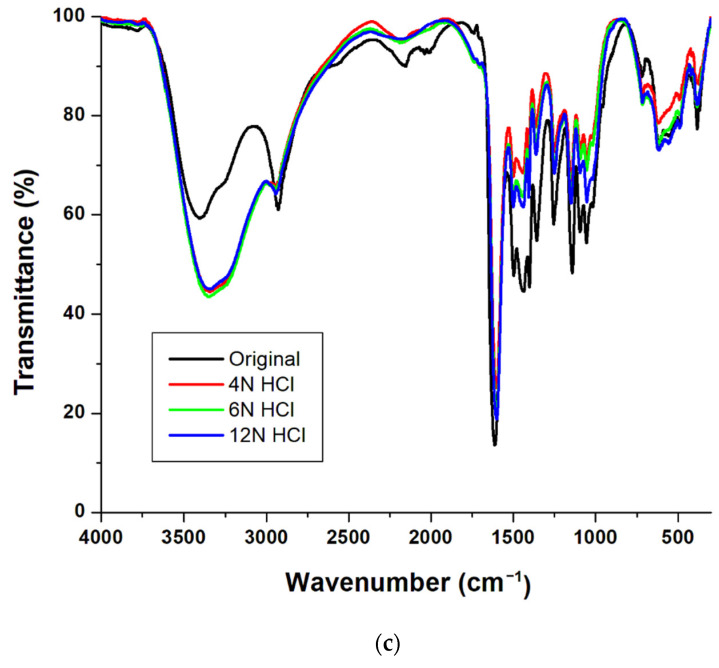
IR spectra of amino-Li-resin after overnight treatment in (**a**) 100% DBU, BF_3_-OEt_2_ and untreated resin, (**b**) 100% TFA, 50% NaOH in H_2_O, 20% piperidine in DMF and untreated resin, and (**c**) 4N, 6N, and 12N HCl and untreated resin.

The polyacrylamide-based resin was examined by infrared spectroscopy (IR) after overnight treatment in a range of reagents used in SPPS, including acids and bases. Diagnostic peaks corresponding to the amide moiety observed between 3100 cm^−1^ and 3500 cm^−1^ for the N–H stretch and between 1610 cm^−1^ and 1700 cm^−1^ for the C=O stretch was used to evaluate its stability. Amino-Li-resin was relatively stable in organic reagents (Figure 4a), as well as in hydrochloric acid (HCl) at all the concentrations tested (Figure 4c). It retained its chemical stability after treatment in 20% piperidine in DMF and 100% TFA used for deprotection and global cleavage, respectively. All spectra results showed no observable changes between the treated resins and the untreated ones (designated as original in Figure 4a–c); thus, confirming its chemical stability and broad compatibility.

### 3.3. Swelling

**Figure 5:**
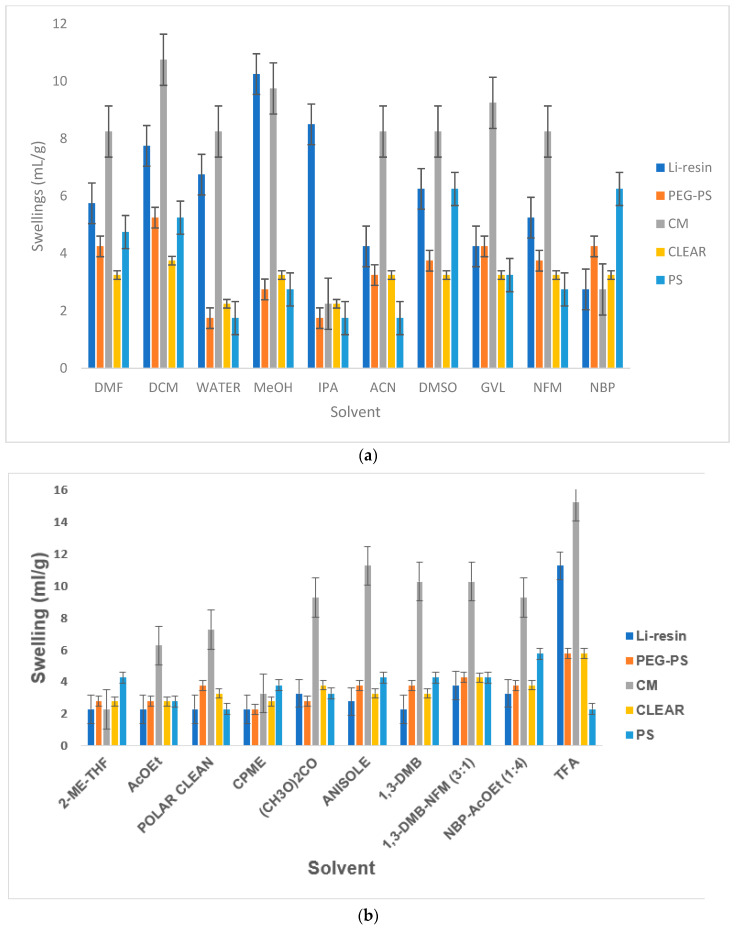
(**a**,**b**) Swelling of resins in different solvents.

A primary consideration for the suitability of any given resin in SPPS is its swelling capacity. This is an important property since it indicates the availability of the resin to interact with a variety of solvents and common reagents. The swelling capacity of amino-Li-resin was compared with that of PS, PEG-PS, CM, and CLEAR resins in a broad range of solvents, including protic and non-protic polar solvents, and also green solvents (Table 1, Figure 5a,b).

Amino-Li-resin swelled better than PS and PEG-PS but a little less than CM in DMF (#1), which is the most used solvent for SPPS both in research and industry. Although DMF is not a green solvent, it can be beneficial since it brings about enhanced swelling with respect to PS, but without a disproportionate increase in the consumption of solvents in the different steps of SPPS, particularly in the washes. In DCM (#2), which is considered more hazardous than DMF, the same trend was observed. Generally, amino-Li-resin swelled well in protic polar solvents such as H_2_O (#3), MeOH (#4), and IPA (#5), this making this support highly suitable for biochemical assays involving these solvents [49]. Interestingly, Li-resin swelled well in ACN (#6), which is a solvent specifically indicated to be useful for difficult couplings [9,18].

With the advent of the mandate of greening all chemical processes, including SPPS, resin compatibility with green solvents is a plus [50]. In this regard, amino-Li-resin showed good swelling in DMSO (#7), GVL (#8), and NFM (#9), the latter considered a green analog of DMF. However, its swelling capacity in NBP (#10) was lower than in the previous solvents. In other green solvents (#11-14), including two aromatics (anisole #16 and 1,3-DMB #17), the resin showed the same degree of swelling. Furthermore, swelling in mixtures of green solvents [1,3-DMB-NFM (3:1) #18 and NBP-AcOEt (1:4) #19] was acceptable in both cases. Finally, in TFA (#20), which is the reagent used for the final global deprotection and cleavage, the swelling of amino-Li-resin was lower than in CM, which is also good for avoiding extra consumption of this reagent.

**Figure 6:**
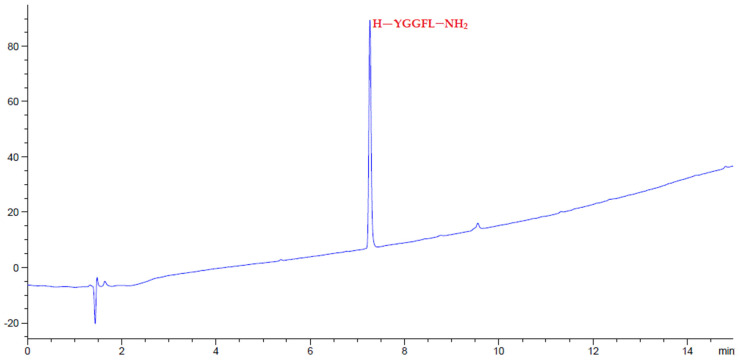
HPLC chromatograms of crude peptides H-YGGFL-NH_2_ after precipitation.

**Figure 7:**
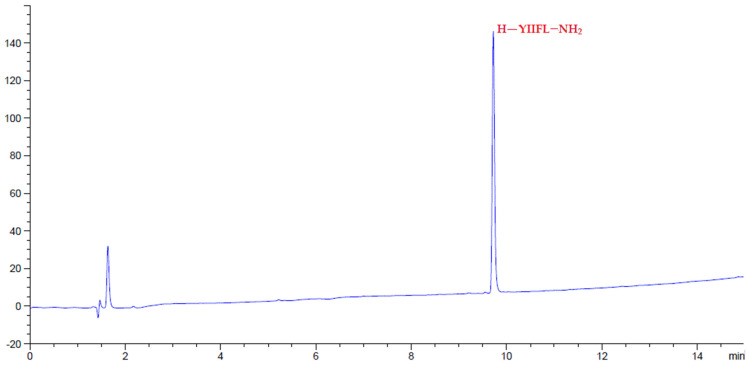
HPLC chromatograms of crude peptides H-YIIFL-NH_2_ after precipitation.

**Figure 8:**
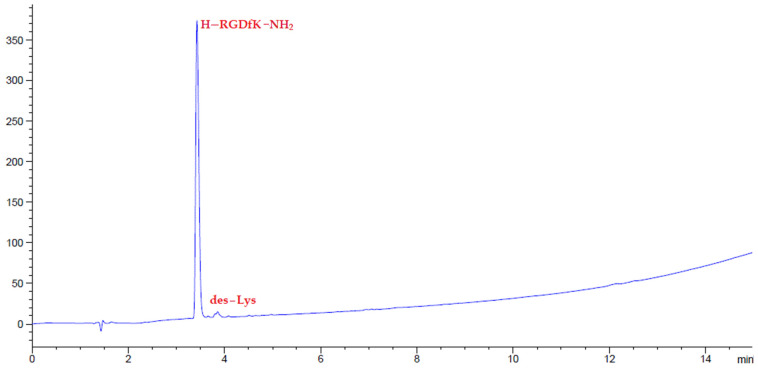
HPLC chromatograms of crude peptides H-RGDfK-NH_2_ after precipitation.

Given the interesting physical characteristics of amino-Li-Resin, it was used for the synthesis of selected model peptides using the standard fluorenylmethoxycarbonyl (Fmoc)/tert-butyl (tBu) protocol, by means of *N*,*N*’-diisopropylcarbodiimide (DIC)-OxymaPure as coupling method at room temperature. We started the evaluation with the manual synthesis of the model Leu-enkephalin peptide (H-YGGFL-NH_2_) using the Rink-amide-Li-resin. At the end of the peptide elongation, the pentapeptide was successfully cleaved with TFA-H_2_O-TIS (95:2.5:2.5), precipitated with ether, lyophilized, and analyzed on HPLC, giving 100% purity (Figure 6). The efficacy of the Rink-amide-Li-resin was further examined in a more hindered pentapeptide where the two Gly residues were replaced by Ile (H-YIIFL-NH_2_) and again afforded 100% purity, as reflected by the HPLC analysis (Figure 7). The RGD peptide (H-RGDfK-NH_2_), which gave 97% purity from HPLC analysis (Figure 8) is comparable with RGD analogues prepared with 2-chlorotrityl resin [51].

**Figure 9:**
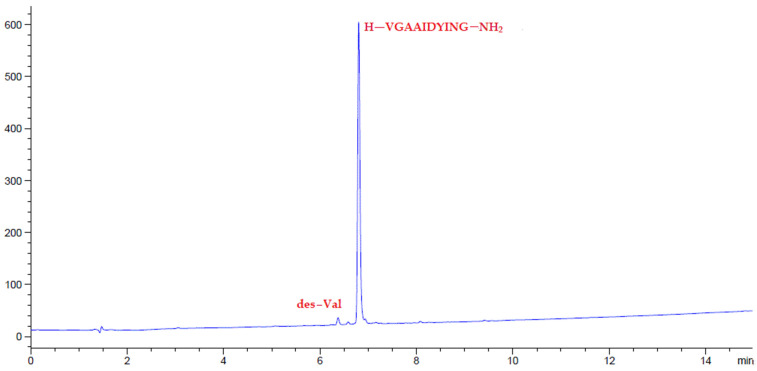
HPLC chromatograms of crude peptides H-VGAAIDYING-NH_2_ after precipitation.

Following the excellent purity of the model pentapeptides obtained from HPLC, we proceeded to synthesize other demanding peptide sequences like ^66^Gly-^65−74^ACP (H-VGAAIDYING-NH_2_), which gave a purity of >95%, with the most important impurity being the deletion of Val (Figure 9).

**Figure 10:**
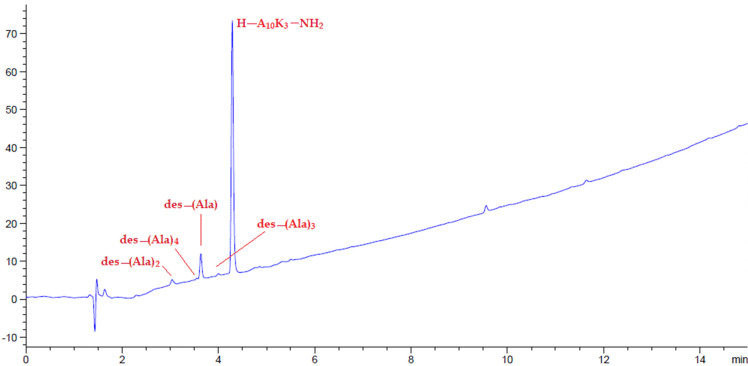
HPLC chromatograms of crude peptides H-(Ala)_10_(Lys)_3_-NH_2_.

Amino-Li-resin was also tested with a modification of the difficult model peptide decaalanine [(Ala)_10_] [52]. The modified [(Ala)_10_] described herein is a 13-mer sequence (H-Ala-Ala-Ala-Ala-Ala-Ala-Ala-Ala-Ala-Ala-Lys-Lys-Lys-NH_2_) containing ten consecutive Ala and three Lys at the N-terminal added to help the solubility. Figure 10 shows the HPLC chromatogram of the crude peptide after mini cleavage to give the title compound with 87.7% purity, accompanied by the deletion of one, two, three, and four Ala as identified by mass spectroscopy. More serious deletions were observed for this difficult sequence by using other synthesis resins. The purity of this test synthesis by using amino-Li-resin is significantly better in comparison with the data obtained from using other resins [52].

## 4. Conclusions and Perspectives

Amino-Li-resin is a new generation of polyacrylamide resin that is highly suitable for solid-phase chemistry. It has a high swelling capacity in a broad range of solvents, preferably in polar ones, but including those most used in SPPS and some of the green solvents used to work in a more sustainable environmental ecosystem. Of note, amino-Li-resin swells extremely well in H_2_O and alcohols of low molecular weight, thus making it highly suitable for biochemical applications such as the development of supports for affinity chromatography and the preparation of supported chemical libraries for on-resin screening. The rather highly cross-linked matrix confers the resin with excellent mechanical stability, which, along with its stability in the presence of a wide range of chemical reagents such as acids and bases, makes it particularly suitable for supported solid-phase organic synthesis. Unlike PS and other resins that are marketed in the form of beads, amino-Li-resin is in the form of fibers, thus maximizing surface area. Amino-Li-resin has a high functionalization (0.8 mmol/g), thereby conferring effectiveness in terms of peptide synthesized per gram of initial resin used. The absence of aromatic rings in amino-Li-resin should avoid the formation of π-π interactions, which can favor resin collapse. The appropriateness of amino-Li-resin for SPPS has been demonstrated for the synthesis of several model peptides, including difficult sequences and hindered amino acids. The compatibility of amino-Li-resin with ACN could be very helpful for hindered couplings because it has been demonstrated that ACN is a good solvent for this kind of couplings.

More so, because the resin can swell very well in water, solid phase synthesis using water as solvent, or a co-solvent can be designed and conducted so that the process can be implemented in an environmentally friendly manner. Furthermore, because the resin is stable with extreme acidic or basic conditions, the biomolecules such as peptides can be synthesized and attached permanently to the resin. The resin, which swells well in water, opens a way to be used for affinity chromatography or even for direct immunization using peptide conjugated with the resin [53]. Overall, this novel resin overcomes the shortcomings of polystyrene resin and polyethylene glycol resin. The significance is that the resin can potentially become the resin of choice to replace both polystyrene resin and polyethylene glycol resin, streamlining the solid phase synthesis for its universal use.

## Figures and Tables

**Table 1:** Swelling of the selected resins in green and non-green solvents.

Entry (#)	Solvent	Swelling (mL/g)				
		Li-resin	PS	PEG-PS	CM	CLEAR
1	DMF	5.75	4.75	4.25	8.25	3.25
2	DCM	7.75	5.25	5.25	10.75	3.75
3	H_2_O *	6.75	1.75	1.75	8.25	2.25
4	MeOH	10.25	2.75	2.75	9.75	3.25
5	IPA *	8.5	1.75	1.75	2.25	2.25
6	ACN	4.25	1.75	3.25	8.25	3.25
7	DMSO *	6.25	6.25	3.75	8.25	3.25
8	GVL *	4.25	3.25	4.25	9.25	3.25
9	NFM *	5.25	2.75	3.75	8.25	3.25
10	NBP *	2.75	6.25	4.25	2.75	3.25
11	2-Me-THF *	2.25	4.25	2.75	2.25	2.75
12	AcOEt *	2.25	2.75	2.75	6.25	2.75
13	Polar Clean *	2.25	2.25	3.75	7.25	3.25
14	CPME *	2.25	3.75	2.25	3.25	2.75
15	(CH_3_O)_2_CO	3.25	3.25	2.75	9.25	3.75
16	Anisole *	2.75	4.25	3.75	11.25	3.25
17	1,3-DMB *	2.25	4.25	3.75	10.25	3.25
18	1,3-DMB-NFM (3:1) *	3.75	4.25	4.25	10.25	4.25
19	NBP-AcOEt (1:4) *	3.25	5.75	3.75	9.25	3.75
20	TFA	11.25	2.25	5.75	15.25	5.75

MeOH, Methanol; IPA, Isopropanol; ACN, Acetonitrile; DMSO, Dimethyl sulphoxide; GVL, gamma-Valerolactone; NFM, *N*-Formylmorpholine; NBP, *N*-Butyl-2-pyrrolidone; 2-Me-THF, 2-methyltetrahydrofuran; AcOEt, Ethyl acetate; CPME, cyclopentyl methyl ether; (CH_3_O)_2_CO, Dimethylcarbonate; 1,3-DMB, 1,3-Dimethylbenzene; TFA, Trifluoroacetic acid; * Green Solvent.

## Data Availability

The data presented in this study are available within the article.
